# Smoky Chimneys

**Published:** 1873-06

**Authors:** 


### SMOKY CHIMNEYS.

Let the "throat" of the chimney, the part just above the fireplace, be small—say six inches by twelve; then immediately after construct it for some two feet high in such a way as to give a sudden bulge of a foot or more broad, and as much longer as possible; then at the end of two feet, gradually contract the chimney passage to any desired size. Thus built no chimney can smoke.

